# Deep learning based automated HER2 score prediction using immunohistochemistry histopathological images: a dual-center study

**DOI:** 10.3389/fphys.2026.1868102

**Published:** 2026-06-25

**Authors:** Juan Ma, Lijun Song, Mireguli Damaola, Mei Zhang, Yi You, Xiongling Tian, Abudushalamu Abulaiti, Diliaremi Aihaiti, Mayidili Nijiati

**Affiliations:** 1 Medical Imaging Center, Xinjiang Medical University Affiliated Fourth Hospital, Urumqi, China; 2 Department of Radiology, The First People's Hospital of Kashi (Kashgar) Prefecture, Kashi, China; 3 Department of Deepwise AI lab, Hangzhou Deepwise & League of PHD Technology Co., Ltd, Hangzhou, China; 4 Department of Ultrasonography, Shule County People’s Hospital, Kashi, China; 5 Department of Ultrasonography, Xinjiang Key Laboratory of Artificial Intelligence assisted Imaging Diagnosis, Kashi, China

**Keywords:** artificial intelligence, breast cancer, deep learning, HER2 status, immunohistochemistry histopathological images

## Abstract

**Background:**

HER2 is a critical prognostic biomarker in breast cancer and associated with aggressive tumor biology. Current IHC scoring is subjective and labor-intensive. Deep learning has demonstrated success in histopathological image analysis, yet HER2 IHC automation remains underexplored. External-center validation is essential to establish clinical credibility and demonstrate robustness across diverse institutional practices and imaging protocols.

**Methods:**

This dual-center retrospective study analyzed 135 HER2 IHC whole-slide images from 118 breast cancer patients labeled as 1+, 2+, or 3+ by standard clinical criteria. Two board-certified pathologists manually annotated tumor-enriched ROIs, which were tiled into non-overlapping 512x512 patches; tiles with >60% white background were excluded. Patches were harmonized using a modified Macenko color normalization and augmented during training. Six pretrained deep learning models (AlexNet, VGG16, ResNet34, DenseNet121, Inception, Swin Transformer) were trained with patient-level splits and evaluated on an independent test set using macro-averaged AUC and complementary metrics.

**Results:**

The cohort included 118 patients with comparable age and largely similar baseline imaging/pathologic characteristics across groups, although clinical symptoms and lymph node status differed. On the independent test set, all models showed good discrimination for three-class HER2 grading, with AlexNet performing best (macro-AUC 0.971), followed by VGG16 (0.967). For AlexNet, per-class AUCs were 0.980 (1+), 0.955 (2+), and 0.979 (3+); most errors occurred between adjacent grades (1+/2+, 2+/3+). Grad-CAM highlighted strongly stained tumor regions driving predictions.

**Conclusion:**

A deep learning framework showed encouraging patch-level performance for three-class HER2 IHC score prediction in a pooled dual-center retrospective cohort. This approach may assist pathologists by improving scoring consistency and identifying borderline or low-confidence cases that require careful review and, when clinically indicated, confirmatory testing.

## 1 Introduction

HER2 (human epidermal growth factor receptor 2) is a critical prognostic and predictive biomarker in breast cancer, with profound implications for patient stratification and therapeutic decision-making (Tarighati et al., 2023). Approximately 15–20% of breast cancers exhibit HER2 overexpression or gene amplification, which is associated with aggressive tumor biology, increased proliferation rates, and enhanced metastatic potential (Yoon and Oh, 2024). The advent of HER2-targeted therapies has substantially improved outcomes for HER2-positive patients, transforming breast cancer from a uniformly poor prognosis to a manageable disease with significantly prolonged survival (Oh and Bang, 2020; Yoon and Oh, 2024). Consequently, accurate and reproducible HER2 assessment is essential for appropriate patient selection and optimal therapeutic allocation. Current clinical practice relies on immunohistochemistry (IHC) scoring combined with fluorescence *in situ* hybridization (FISH) confirmation for equivocal cases, following standardized guidelines established by the American Society of Clinical Oncology (ASCO) and College of American Pathologists (CAP) (Marchiò et al., 2021). However, this conventional workflow is labor-intensive, time-consuming, and dependent on subjective pathologist interpretation, creating significant bottlenecks in clinical throughput and introducing diagnostic variability.

Inter-observer and intra-observer variability in HER2 IHC scoring represents a substantial clinical challenge, with reported concordance rates ranging from 60–85% among experienced pathologists. This diagnostic discordance is particularly pronounced in borderline cases (IHC 2+), where approximately 30–40% of cases require FISH reflex testing to resolve ambiguity (Zaakouk et al., 2023). Sources of variability include differences in antibody batches, chromogen development protocols, scanner calibration, and subjective interpretation of staining intensity and distribution patterns. Such inconsistencies can lead to misclassification, delayed treatment initiation, and suboptimal therapeutic outcomes. Furthermore, the increasing volume of digital pathology data and the growing demand for rapid turnaround times in clinical laboratories have created urgent pressure to develop standardized, objective, and scalable approaches to HER2 assessment (Tewary and Mukhopadhyay, 2021). Digital pathology platforms and computational image analysis offer promising solutions to address these limitations by enabling quantitative, reproducible, and high-throughput analysis of histopathological images.

Recent advances in deep learning and convolutional neural networks (CNNs) have demonstrated remarkable success in automated histopathological image analysis, including cancer detection, grading, subtyping, and biomarker prediction (Kosaraju et al., 2022). Landmark studies have shown that well-trained deep learning models can match or exceed pathologist performance on diverse diagnostic tasks, including prostate cancer grading (Xiang et al., 2023), colorectal cancer staging (Ben Hamida et al., 2021). Vision transformer architectures and hybrid models incorporating self-attention mechanisms have further expanded the capability to capture long-range dependencies and hierarchical feature representations in complex tissue images (Xu et al., 2024). However, the application of deep learning to HER2 IHC automation remains relatively underexplored compared to H&E-stained histology. Existing studies on HER2 quantification have primarily focused on pixel-level intensity measurements or simple statistical features (Bannier et al., 2024), lacking the sophistication of modern deep learning approaches. Moreover, most published work has been conducted within single-center settings, raising concerns about generalization across diverse staining protocols, scanner platforms, and institutional practices (Bae et al., 2023). Multi-center validation is critical to establish clinical credibility and demonstrate robustness to real-world variability in image acquisition and preprocessing.

Prior computational approaches for HER2 IHC assessment can be broadly divided into rule-based quantitative image analysis, cell- or membrane-segmentation-based deep learning, and slide- or patch-level classification models. Rule-based methods quantify staining intensity and membrane completeness but may be sensitive to staining variation and require carefully engineered features. Segmentation-based methods, such as HER2Net, provide biologically interpretable analysis of membranes and nuclei but depend on labor-intensive pixel-level annotations and may be difficult to scale across institutions (Saha and Chakraborty, 2018). Patch-level and WSI-level classification approaches are easier to adapt to retrospective cohorts because they can use clinically assigned HER2 scores as labels, but their generalizability requires validation across centers. The present study addresses this latter need by evaluating a standardized patch-based HER2 classification framework in a dual-center cohort.

In this study, we evaluated six representative pretrained deep learning architectures, including conventional convolutional neural networks and a transformer-based model, for automated HER2 IHC score classification from breast cancer whole-slide images collected from two clinical centers. The objective was not to introduce a novel architecture, but to establish a reproducible and clinically feasible benchmark pipeline incorporating pathologist-guided tumor ROI annotation, color normalization, patch-level training, patient-level data splitting, and independent test-set evaluation. By positioning the analysis in a dual-center setting, this study aims to provide evidence for the feasibility and robustness of automated HER2 IHC grading using routinely available clinical WSI data, while identifying limitations that should guide future development of more task-specific HER2 models.

## 2 Methods

### 2.1 Study population and data acquisition

This retrospective study included breast cancer patients from two independent medical centers. A total of 135 immunohistochemistry (IHC) whole-slide images (WSIs) from 118 patients were analyzed, with some patients contributing more than one WSI. HER2 statues histopathologically assessed using standard clinical scoring criteria and categorized into three groups: 1+, 2+, and 3+, which served as the ground truth labels for model training and validation. HER2 IHC scores were interpreted according to the ASCO/CAP HER2 testing guideline criteria. The endpoint of the present model was prediction of the HER2 IHC score category itself, namely 1+, 2+, or 3+, rather than prediction of HER2 gene amplification or final molecular HER2-positive/negative status. Although IHC 2+ cases are considered equivocal in routine clinical practice and generally require confirmatory ISH/FISH testing, ISH/FISH results were not used as labels for model training or evaluation in this study.HER2 IHC scores were evaluated by two board-certified pathologists according to standard clinical HER2 IHC scoring criteria. In cases with discrepant interpretations, the slides were jointly reviewed, and a final consensus score was assigned. The final consensus HER2 IHC score at the WSI/case level served as the reference label for model training and evaluation. HER2–0 cases were not included in the present analysis because sufficient HER2–0 IHC WSIs were not available in the retrospective digital pathology cohort during model development. Therefore, this study focused on three-class HER2 IHC score prediction among 1+, 2+, and 3+ cases. Ethical approval was obtained from institutional ethical review board and due to retrospective nature of this study, written informed consent was waived.

### 2.2 Image preprocessing

#### 2.2.1 Study design

**Figure 1:**
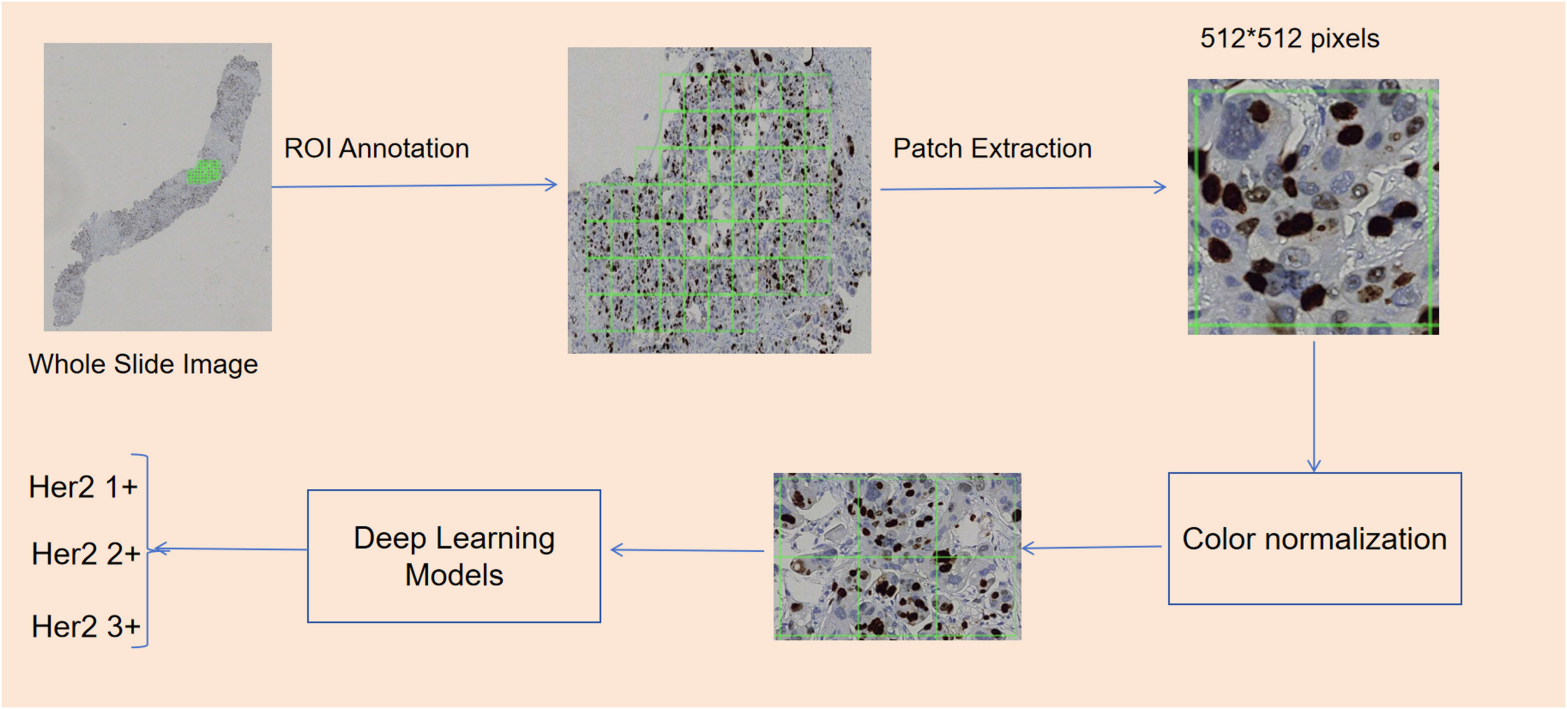
Workflow of the study.

The analytical workflow proceeded as shown in **Figure 1**, IHC images were first reviewed by two board-certified pathologists who manually annotated tumor-enriched regions of interest (ROIs) to exclude stromal and necrotic areas. Within these annotated ROIs, IHC images were systematically tiled into non-overlapping 512×512 pixel patches at 512-pixel stride intervals. Patches containing >60% white background were excluded to ensure data quality. The resulting high-quality patches underwent color normalization using a modified Macenko method to harmonize staining variability across centers and imaging protocols. Normalized patches were then used to train and evaluate six deep learning architectures for three-class HER2 classification (1+, 2+, 3+). Model performance was assessed on an independent test set using macro-averaged AUC as the primary metric, supplemented by per-class accuracy, precision, recall, F1-score, and confusion matrices.

#### 2.2.2 Color normalization

Variability in IHC staining intensity and color, arising from factors such as antibody batch differences, chromogen development time, and scanner parameters, can potentially confound quantitative analysis. To harmonize color representation across WSIs, a modified Macenko method was applied for stain separation and color normalization. This approach established a standardized color space that mitigated non-biological variability and facilitated consistent downstream feature extraction.

#### 2.2.3 Region of interest annotation

Two board-certified pathologists reviewed all HER2 IHC WSIs using a multimodal research platform (https://keyan.deepwise.com,version 3.1.2) and manually delineated tumor-enriched ROIs. ROI annotation focused on representative invasive carcinoma areas suitable for HER2 IHC assessment. Non-tumor regions, stromal-dominant areas, necrosis, tissue folds, staining artifacts, benign epithelium, DCIS-only areas, and background regions were excluded whenever possible. When there were differences in ROI selection, the slides were jointly reviewed and the final annotated ROIs were determined by consensus.

#### 2.2.4 Patch extraction and quality control

Within annotated tumor ROIs, WSIs were tiled into non-overlapping patches of 512×512 pixels with a stride of 512 pixels to systematically cover tumor heterogeneity. Patches with more than 60% white background were excluded to ensure data quality. Following this procedure, 3,767 high-quality patches were obtained for model development and evaluation. Each extracted patch inherited the HER2 IHC score label of its corresponding WSI/case. Thus, patch-level labels were derived from slide-level clinical HER2 IHC scores rather than from independent patch-level pathological scoring.

#### 2.2.5 Data augmentation

To increase training sample diversity and enhance model robustness, multiple augmentation techniques were applied randomly during training, including random cropping to mimic variation in field of view and magnification; horizontal and vertical flipping to leverage tissue symmetry; elastic deformation simulating mechanical distortions in slide preparation; random rotations addressing orientation invariance; color jitter adjusting brightness and contrast to mimic staining variability; and addition of Gaussian noise to replicate scanner-induced artifacts. These combined augmentations improve the model’s generalization to diverse image acquisition conditions.

### 2.3 Model development, selection and training

A total of 3,767 high-quality patch-level samples were generated from the 135 WSIs following ROI annotation, patch extraction, background exclusion, and color normalization. It should be noted that WSIs from both centers were pooled together before these steps, and no center-level separation was applied during patch generation. To avoid data leakage, dataset splitting was performed at the patient level rather than at the patch level. The dataset was divided into training, validation, and testing sets at a ratio of 70%, 15%, and 15%, respectively, preserving patient independence across subsets. This resulted in 2,644 patches for training, 564 patches for validation, and 559 patches for independent testing. Specifically, based on unique patient IDs, all WSIs from the same patient were assigned exclusively to one of the three subsets, ensuring no patient overlap across subsets. Thus, no patient contributed to more than one subset, and this design ensures that model evaluation reflects generalization to unseen patients rather than memorization of slide-level features.

#### 2.3.1 Model selection

Six representative pretrained deep learning architectures were selected to benchmark different categories of models under an identical training and evaluation pipeline: AlexNet, VGG16, ResNet34, DenseNet121, Inception, and Swin Transformer. AlexNet and VGG16 were included as classical convolutional neural network baselines, representing relatively simple and deeper feed-forward CNN architectures, respectively. ResNet34 and DenseNet121 were selected to represent residual and densely connected convolutional architectures, which are designed to mitigate vanishing gradients and enhance feature reuse. Inception was included because of its multi-scale convolutional feature extraction capability. Swin Transformer was evaluated as a representative vision transformer architecture using hierarchical shifted-window self-attention to capture both local and long-range contextual information. The purpose of this comparison was to assess the feasibility and relative performance of widely available architectures for HER2 IHC grading in a dual-center setting, rather than to propose a novel network design.

All models were initialized with ImageNet-pretrained weights for transfer learning to accelerate convergence and leverage general visual feature representations. The final classification layers of each network were modified to output three HER2 IHC categories: 1+, 2+, and 3 +. All models were trained using the same experimental settings to ensure fair comparison. Cross-entropy loss was used as the objective function, and optimization was performed using the Adam algorithm with an initial learning rate of 0.001. The batch size was set to 4. Training was allowed for a maximum of 50 epochs, and early stopping was applied if the validation loss did not improve for ten consecutive epochs. All hyperparameters were tuned solely based on the training and validation sets. The validation set was also used for early stopping and model checkpoint selection. The model with the best validation performance was retained for final evaluation on the independent test set. The test set remained completely untouched throughout the entire model development process (including hyperparameter tuning, training, and model selection) and was used only once to report the final performance.

### 2.4 Performance evaluation

Model discrimination was primarily assessed by area under the macro-averaged receiver operating characteristic curve (macro-averaged AUC) on the independent test set, which treats all classes equally regardless of sample size. Micro-average AUC was also computed to provide a complementary view of overall performance aggregated across all predictions. To further evaluate classification robustness, we examined precision-recall (PR) curves and reported per-class accuracy, precision, recall, and F1-score. A confusion matrix was generated to visualize inter-class misclassification patterns across HER2 categories. All performance metrics, including ROC curves, precision-recall curves, confusion matrices, accuracy, precision, recall, F1-score, and AUC, were calculated at the patch level. Slide-level HER2 IHC labels were assigned to all patches extracted from the corresponding annotated tumor ROIs. No slide-level or patient-level aggregation was performed in the current analysis. For performance estimation, 95% confidence intervals (CIs) were calculated using a nonparametric bootstrap approach. All of the above metrics were calculated at the patch level. Interpretability was explored through Gradient-weighted Class Activation Mapping (Grad-CAM) to identify salient image regions driving predictions.

## 3 Results

### 3.1 Dataset characteristics:

Patient characteristics. A total of 135 HER2 IHC WSIs from 118 patients were included from two medical centers. Among these, Center 1 contributed 101 patients and 110 WSIs, and Center 2 contributed17 patients and 25WSIs (HER2 1+, n=41; HER2 2+, n=57; HER2 3+, n=20; **Table 1**). Median age was comparable across groups [50.0 (44.0-58.0), 51.0 (44.0-58.0), and 54.5 (49.5-57.5) years for HER2 1+, 2+, and 3+, respectively; *P* = 0.731]. Clinical symptoms differed by HER2 category, with redness/pain more frequent in the HER2 2+ group (42.1%) than in HER2 1+ (17.1%) and HER2 3+ (5.0%) (*P* = 0.001), and lymph node status also varied significantly (*P* = 0.019), showing a higher proportion of nodal involvement in HER2 2+/3+ compared with HER2 1 +. Histopathologic subtype distribution was similar across groups (predominantly invasive ductal carcinoma: 80.5%-85.0%; *P* = 0.931), and pathologic grade did not differ significantly (*P* = 0.149).

**Table 1:** Baseline clinical, imaging, and pathological characteristics were summarized according to HER2 IHC score group.

Variable	HER2+ (n=41)	HER2++ (n=57)	HER2+++ (n=20)	P value
Age	50.00 (44.00-58.00)	51.00 (44.00-58.00)	54.50 (49.50-57.50)	0.731
Redness_Pain				0.001
Yes	7 (17.1%)	24 (42.1%)	1 (5.0%)	
No	34 (82.9%)	33 (57.9%)	19 (95.0%)	
Nipple_Discharge				0.563
Yes	3 (7.3%)	3 (5.3%)	0 (0.0%)	
No	38 (92.7%)	54 (94.7%)	20 (100.0%)	
Laterality				0.559
Left	18 (43.9%)	19 (33.3%)	8 (40.0%)	
Right	23 (56.1%)	38 (66.7%)	12 (60.0%)	
BPE				0.920
Minimal	24 (58.5%)	32 (56.1%)	10 (50.0%)	
Mild	13 (31.7%)	19 (33.3%)	9 (45.0%)	
Moderate	4 (9.8%)	6 (10.5%)	1 (5.0%)	
Marked	0 (0.0%)	0 (0.0%)	0 (0.0%)	
FGT				0.231
Almost entirely fatty	5 (12.2%)	7 (12.3%)	5 (25.0%)	
Scattered fibroglandular tissue	14 (34.1%)	17 (29.8%)	7 (35.0%)	
Heterogeneously dense	14 (34.1%)	20 (35.1%)	6 (30.0%)	
Extremely dense	8 (19.5%)	13 (22.8%)	2 (10.0%)	
Quadrant				0.267
Upper outer	13 (31.7%)	20 (35.1%)	6 (30.0%)	
Lower outer	2 (4.9%)	9 (15.8%)	7 (35.0%)	
Upper inner	14 (34.1%)	14 (24.6%)	2 (10.0%)	
Lower inner	5 (12.2%)	7 (12.3%)	3 (15.0%)	
Retroareolar	3 (7.3%)	3 (5.3%)	1 (5.0%)	
Central	4 (9.8%)	4 (7.0%)	1 (5.0%)	
Focus_num				0.151
Single	27 (65.9%)	42 (73.7%)	10 (50.0%)	
Multiple	14 (34.1%)	15 (26.3%)	10 (50.0%)	
X_Margin				0.458
Well-defined	3 (7.3%)	3 (5.3%)	1 (5.0%)	
Obscured	1 (2.4%)	1 (1.8%)	2 (10.0%)	
Lobulated	13 (31.7%)	24 (42.1%)	5 (25.0%)	
Ill-defined	18 (43.9%)	16 (28.1%)	7 (35.0%)	
Spiculated	6 (14.6%)	13 (22.8%)	5 (25.0%)	
X_Shape				0.488
Round	1 (2.4%)	5 (8.8%)	2 (10.0%)	
Oval	14 (34.1%)	14 (24.6%)	7 (35.0%)	
Irregular	26 (63.4%)	38 (66.7%)	11 (55.0%)	
Nipple_Retract				0.501
Yes	3 (7.3%)	9 (15.8%)	2 (10.0%)	
No	38 (92.7%)	48 (84.2%)	18 (90.0%)	
Skin_Thick				0.302
Yes	3 (7.3%)	8 (14.0%)	4 (20.0%)	
No	38 (92.7%)	49 (86.0%)	16 (80.0%)	
Pectoral_Inv				1.000
Yes	1 (2.4%)	2 (3.5%)	0 (0.0%)	
No	40 (97.6%)	55 (96.5%)	20 (100.0%)	
ChestWall_Inv				1.000
Yes	1 (2.4%)	1 (1.8%)	0 (0.0%)	
No	40 (97.6%)	56 (98.2%)	20 (100.0%)	
LymphNode				0.019
No	31 (75.6%)	30 (52.6%)	8 (40.0%)	
Single	6 (14.6%)	18 (31.6%)	7 (35.0%)	
Multiple	4 (9.8%)	9 (15.8%)	5 (25.0%)	
Path_Grade	5.00 (4.00-5.00)	5.00 (4.75-5.00)	6.00 (6.00-6.00)	0.149
Pathology				0.931
Ductal carcinoma in situ	4 (9.8%)	7 (12.3%)	3 (15.0%)	
Invasive ductal carcinoma	33 (80.5%)	46 (80.7%)	17 (85.0%)	
Lobular carcinoma in situ	2 (4.9%)	1 (1.8%)	0 (0.0%)	
Invasive lobular carcinoma	2 (4.9%)	3 (5.3%)	0 (0.0%)	
Her2				0.000
1+	41 (100.0%)	0 (0.0%)	0 (0.0%)	
2+	0 (0.0%)	57 (100.0%)	0 (0.0%)	
3+	0 (0.0%)	0 (0.0%)	20 (100.0%)	

### 3.2 Model performance comparison:

All reported model performance results represent patch-level classification performance on the training, validation, and independent test sets. All six deep learning architectures demonstrated strong discriminative capacity for three-class HER2 expression grading (1+, 2+, 3+) on the independent test set. Among the evaluated models, AlexNet achieved the highest macro-averaged AUC of 0.9710 on the test set, followed by VGGNet16 (0.9670), DenseNet121 (0.9100), ResNet34 (0.7960), Swin Transformer (0.8270), and InceptionNet (0.872) (**Table 2**). Notably, the relatively shallow AlexNet architecture achieved slightly better performance than deeper models incorporating residual connections (DenseNet) or self-attention mechanisms (Swin Transformer), although the differences were modest. This may suggest that for HER2 IHC classification, the spatially localized, staining-intensity-driven features can be effectively captured by conventional convolutional hierarchies without necessarily requiring very deep architectures or long-range dependency modeling. However, it should be noted that deeper models still achieved competitive results, and their performance might further improve with larger datasets.

**Table 2:** Model performance metrics for HER2 classification.

Models	Set	AUC	Precision	Recall	Accuracy	F1
AlexNet	training	0.977 [0.972-0.982]	0.898 [0.887-0.910]	0.898 [0.877-0.917]	0.898 [0.890-0.907]	0.897 [0.887-0.907]
	validation	0.977 [0.965-0.988]	0.901 [0.877-0.923]	0.895 [0.850-0.940]	0.894 [0.873-0.916]	0.895 [0.867-0.920]
	testing	0.971 [0.959-0.984]	0.851 [0.823-0.880]	0.862 [0.810-0.913]	0.864 [0.843-0.886]	0.854 [0.827-0.883]
Resent34	training	0.955 [0.947-0.963]	0.856 [0.840-0.870]	0.860 [0.840-0.887]	0.863 [0.852-0.875]	0.857 [0.843-0.870]
	validation	0.957 [0.940-0.973]	0.851 [0.823-0.883]	0.845 [0.793-0.900]	0.851 [0.828-0.874]	0.847 [0.820-0.877]
	testing	0.949 [0.929-0.968]	0.820 [0.790-0.850]	0.826 [0.767-0.883]	0.843 [0.817-0.870]	0.823 [0.790-0.853]
Swin Transformer	training	0.949 [0.941-0.958]	0.836 [0.823-0.853]	0.848 [0.823-0.870]	0.845 [0.833-0.857]	0.841 [0.830-0.857]
	validation	0.948 [0.930-0.966]	0.848 [0.817-0.877]	0.857 [0.807-0.907]	0.851 [0.824-0.878]	0.852 [0.823-0.880]
	testing	0.941 [0.921-0.962]	0.832 [0.800-0.863]	0.854 [0.800-0.907]	0.852 [0.827-0.877]	0.841 [0.810-0.870]
DenseNet121	training	0.946 [0.937-0.955]	0.827 [0.813-0.840]	0.838 [0.813-0.863]	0.838 [0.828-0.848]	0.832 [0.820-0.847]
	validation	0.951 [0.933-0.969]	0.839 [0.807-0.870]	0.840 [0.783-0.897]	0.842 [0.816-0.869]	0.839 [0.810-0.870]
	testing	0.952 [0.935-0.970]	0.815 [0.787-0.847]	0.828 [0.767-0.887]	0.835 [0.808-0.862]	0.820 [0.787-0.850]
Inception	training	0.857 [0.840-0.874]	0.723 [0.707-0.740]	0.737 [0.707-0.763]	0.730 [0.717-0.743]	0.722 [0.707-0.737]
	validation	0.866 [0.832-0.900]	0.735 [0.700-0.770]	0.748 [0.683-0.810]	0.743 [0.712-0.775]	0.735 [0.700-0.770]
	testing	0.872 [0.836-0.908]	0.712 [0.673-0.747]	0.743 [0.680-0.807]	0.733 [0.703-0.763]	0.718 [0.680-0.753]
VGG16	training	0.966 [0.959-0.972]	0.868 [0.857-0.880]	0.868 [0.843-0.890]	0.868 [0.857-0.880]	0.867 [0.853-0.880]
	validation	0.965 [0.949-0.980]	0.874 [0.847-0.900]	0.872 [0.823-0.923]	0.876 [0.856-0.896]	0.872 [0.847-0.897]
	testing	0.957 [0.940-0.975]	0.840 [0.810-0.870]	0.855 [0.800-0.907]	0.853 [0.828-0.878]	0.847 [0.820-0.877]

**Figure 2:**
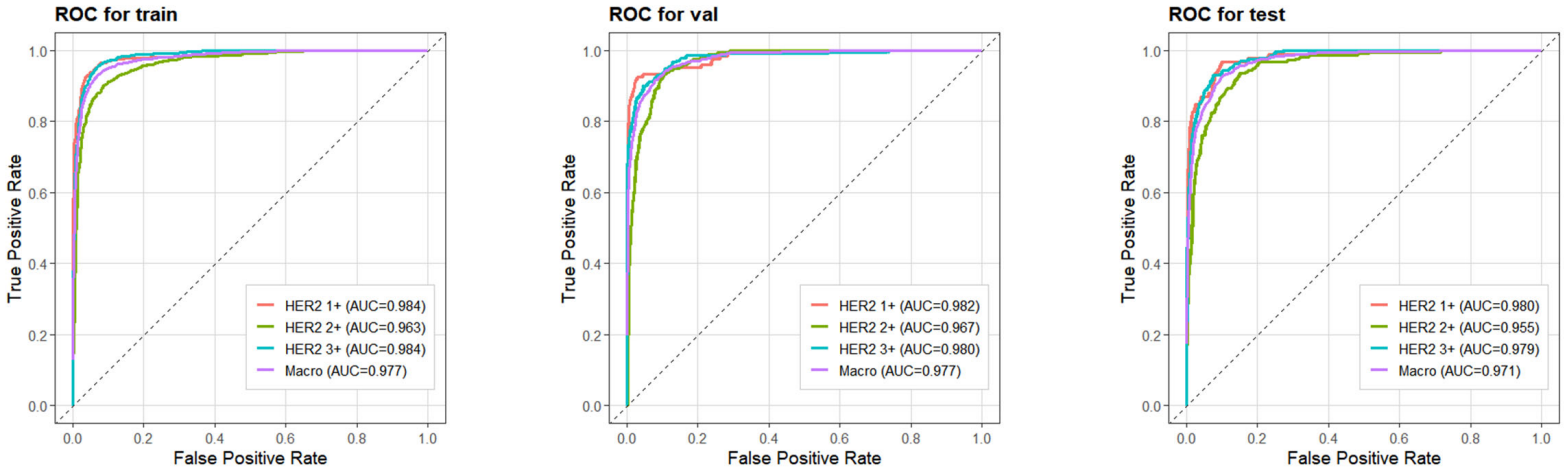
Patch-level receiver operating characteristic curves for HER2 IHC classification in the training, validation, and test sets.

**Figure 3:**
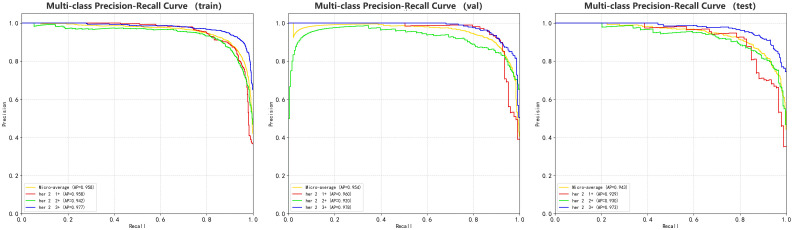
Patch-level precision-recall curves for HER2 IHC classification in the training, validation, and test sets.

For the best-performing model (AlexNet), per-class ROC analysis on the test set yielded AUCs of 0.9800, 0.9550, and 0.9790 for HER2 1+, 2+, and 3+, respectively, with macro-averaged AUC of 0.9710 (**Table 2**; **Figure 2**). Precision-recall analysis corroborated these findings, with per-class average precision (AP) values of 0.9290, 0.9300, and 0.9730 for HER2 1+, 2+, and 3+, respectively, and a micro-averaged AP of 0.9430 (**Table 2**; **Figure 3**). Consistent performance across training, validation, and test sets indicated that the model generalized well without overfitting.

**Figure 4:**
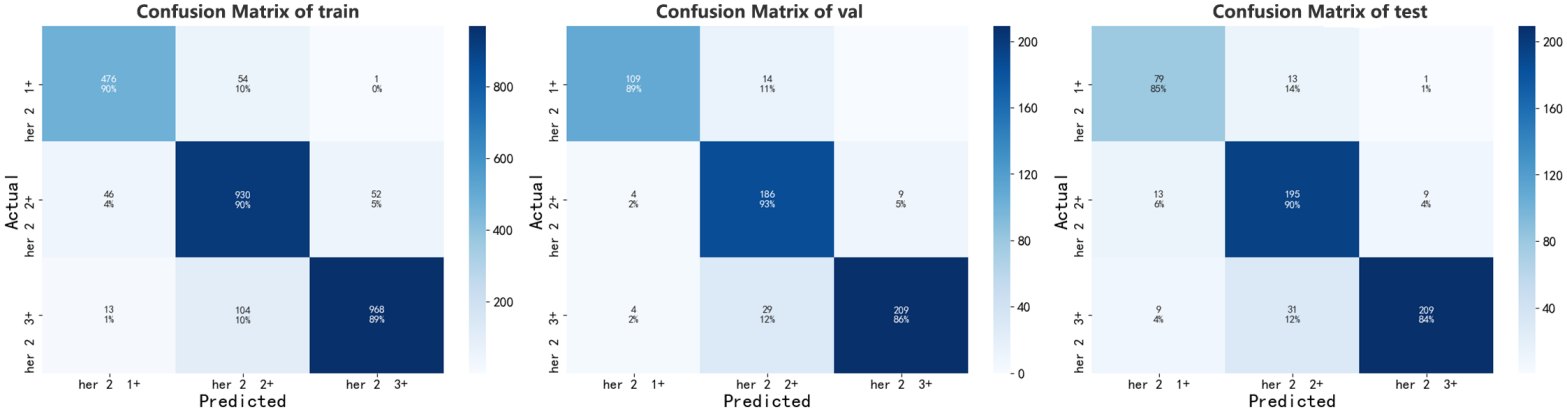
Patch-level confusion matrices for HER2 IHC classification in the training, validation, and test sets.

Confusion matrix analysis revealed that the model correctly classified 85%, 90%, and 84% of HER2 1+, 2+, and 3+ patches on the test set, respectively (**Figure 4**). The predominant misclassification pattern involved HER2 1+ patches being predicted as HER2 2+ (14%), which is clinically expected given the morphological ambiguity at the 1+/2+ boundary. HER2 3+ misclassification was similarly concentrated at the adjacent 2+ category (12%), while HER2 2+ demonstrated the highest classification accuracy, with only 6% and 4% of patches misclassified as 1+ and 3+, respectively.

### 3.3 Visualization and interpretability

**Figure 5:**
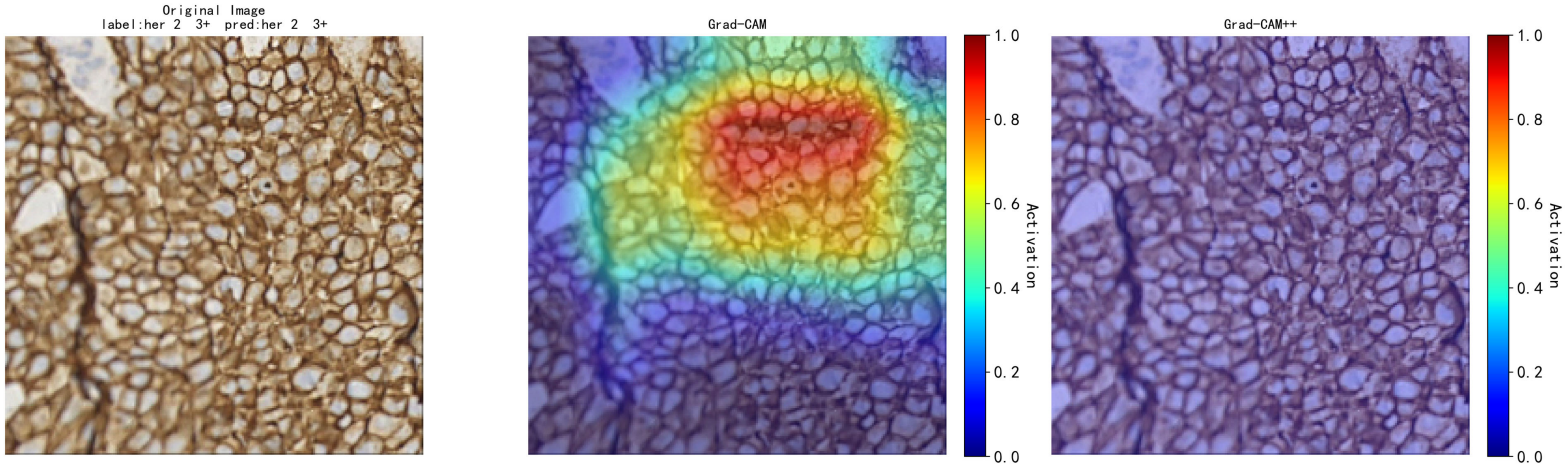
Grad-Cam of accurately classified case.

**Figure 6:**
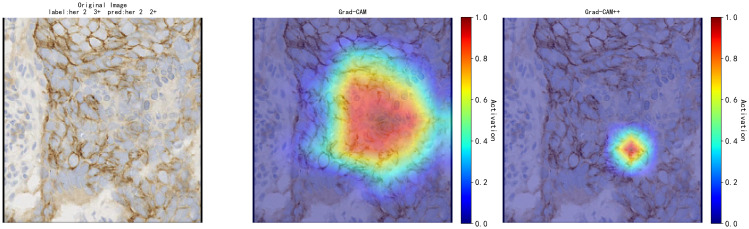
Grad-Cam of misclassified case.

Receiver operating characteristic curves and precision-recall curves validated the superior discriminative ability of AlexNet on the held-out test cohort. Grad-CAM heatmaps localized regions of strongest model activation, predominantly corresponding to areas with intense HER2 staining and dense tumor morphology. (**Figures 5**, **6**). These findings provide qualitative confirmation that the model bases decisions on biologically relevant histological features.

## 4 Discussion

Accurate determination of HER2 status by immunohistochemistry (IHC) is pivotal for therapeutic decision-making in breast cancer, yet routine scoring remains labor-intensive and susceptible to inter- and intra-observer variability, particularly for equivocal 2+ cases that often require reflex FISH testing. In this dual-center retrospective study, we developed an automated HER2 scoring framework using deep learning on IHC whole-slide images, aiming to improve objectivity and scalability of assessment. Tumor-enriched regions were manually delineated by two board-certified pathologists, and within these ROIs slides were tiled into 512x512 patches with exclusion of high-background tiles to ensure image quality. To mitigate staining and scanner variability between institutions, patches were standardized using a modified Macenko color normalization approach and augmented during training to enhance generalization. We benchmarked six representative architectures spanning classical CNNs and modern designs (AlexNet, VGG16, ResNet34, DenseNet121, Inception, and Swin Transformer) for three-class classification (1+, 2+, 3+) using a patient-level train/validation/test split, and evaluated performance primarily by macro-averaged AUC on an independent test set.

Our work should be interpreted in the context of previous studies on automated HER2 image analysis. Saha and Chakraborty proposed HER2Net (Saha and Chakraborty, 2018), a task-specific deep framework for semantic segmentation and classification of cell membranes and nuclei in HER2 breast cancer evaluation. Compared with such segmentation-centered methods, our approach differs in its clinical objective and annotation requirement. HER2Net is designed to explicitly segment cellular structures and therefore requires detailed cell- or membrane-level ground truth annotations. In contrast, our framework uses pathologist-delineated tumor-enriched ROIs and clinical HER2 IHC scores, which are more readily available in routine retrospective WSI datasets. This makes the proposed workflow easier to implement in clinical data settings where dense pixel-level annotation is impractical. However, segmentation-based approaches may provide better biological interpretability by directly assessing membrane completeness and staining intensity at the cellular level. Future work should therefore investigate hybrid approaches that combine scalable WSI-level learning with task-specific membrane-aware modeling.

The observed performance of deep learning models in HER2 IHC scoring is biologically plausible because the classification task is fundamentally driven by reproducible visual cues at the tumor-cell membrane level. HER2 3+ cases typically exhibit strong, circumferential, and continuous membranous staining across a substantial proportion of tumor cells, whereas 1+ cases more often demonstrate faint, incomplete, or barely perceptible membrane staining. In contrast, 2+ slides frequently contain moderate intensity staining with marked intra-tumoral heterogeneity, mixed complete and incomplete membranous patterns, and focal positive regions, making this category intrinsically challenging even for expert observers. These characteristics align well with patch-based learning, where models can aggregate local membrane morphology and staining intensity patterns across multiple tumor regions. Architectures with improved feature propagation can stabilize optimization and reuse discriminative membrane features, while Inception-style multi-scale convolution can capture membrane contours at different spatial resolutions. Swin Transformer further offers hierarchical self-attention that can better model longer-range context and heterogeneity, potentially benefiting borderline 2+ cases.

Prior work on automated HER2 assessment and related prediction tasks generally follows three methodological directions: early pathology-centric systems used explicit, rule-based quantification of staining intensity and membrane completeness with pixel thresholds, handcrafted morphology features, and stepwise pipelines; many later studies, particularly in imaging, were trained and evaluated within a single institution or with limited external validation, potentially inflating performance under homogeneous staining, scanner, and patient distributions (Li et al., 2025). Multimodal designs illustrate that predictive signal may be unevenly distributed across inputs: clinicopathologic variables can dominate recurrence prediction and imaging-only models may underperform, while fusion can yield more balanced operating characteristics when modalities contribute complementary information (Choi et al., 2025). Similarly, combining H&E-derived deep features with clinical variables can improve performance and enable risk stratification, but external testing typically reduces AUC relative to internal validation, emphasizing the need for cross-cohort evaluation and robust normalization (Yang et al., 2022). For biomarker inference from histology, transformer-style attention mechanisms and carefully curated labels can achieve strong binary HER2 discrimination, yet excluding 2+ cases likely increase apparent performance compared with clinically realistic multi-class IHC scoring (Akbarnejad et al., 2025). In HER2 IHC automation specifically, classical pipelines that classify cells and then apply guideline-inspired rules demonstrate that deep learning can surpass handcrafted-feature methods and reach substantial agreement with expert scoring, while discordant cases are often linked to staining heterogeneity that may be clinically informative for flagging difficult slides (Vandenberghe et al., 2017). Evidence from radiology-based HER2 prediction further shows that even simple fusion strategies can improve AUC and markedly shift sensitivity/specificity trade-offs (Fan et al., 2025), and ultrasound studies indicate end-to-end CNNs can outperform radiomics while reducing reliance on precise manual segmentation (Xu et al., 2022). Building on these insights, our work emphasizes end-to-end deep models on HER2 IHC images, benchmarks multiple architectures under a standardized protocol, and evaluates on a dual-center dataset with consistent preprocessing and augmentation to reduce center-specific bias and better estimate real-world generalization.

A realistic deployment scenario is an assistive decision-support tool for pathologists that operates on ROI-based IHC patches within a digital pathology platform, providing a preliminary HER2 score alongside confidence estimates and interpretable heatmaps. In routine workflow, it can triage cases for expedited review, act as a second reader, and specifically flag discordant or low-confidence predictions for closer inspection to improve consistency and reduce time burden. Importantly, it does not replace CAP/ASCO-guided interpretation; rather, it may help identify borderline, discordant, or low-confidence cases that require careful pathologist review and, where appropriate according to clinical guidelines, confirmatory ISH/FISH testing, and standardize initial scoring before final sign-out.

Although the best-performing model achieved a high patch-level macro-AUC on the held-out test set, these results should be interpreted cautiously because of the relatively small retrospective cohort and limited number of test patients. In addition, the test set was generated from a pooled dual-center cohort rather than from a completely center-held-out external validation cohort. Therefore, the current findings should be considered preliminary evidence of feasibility rather than definitive proof of broad clinical generalizability.

## 5 Limitations

Several limitations should be acknowledged. First, we did not directly compare our framework with task-specific models like HER2Net, as the pixel- and cell-level annotations they require were unavailable in our retrospective cohort. Second, our relatively small dual-center dataset requires larger external validation cohorts to confirm generalizability. Third, our patch-level approach presents several constraints. Inheriting slide-level HER2 labels for patch-level training may introduce label noise due to intratumoral staining heterogeneity. Additionally, patch-level bootstrap resampling may slightly underestimate the true uncertainty of model performance due to intra-WSI patch correlations. Finally, without a slide- or patient-level aggregation strategy, our results represent preliminary patch-level evidence rather than clinical slide-level HER2 scoring. Future studies should utilize larger multi-center datasets, cell-level annotations, and comparisons with task-specific or hybrid weakly supervised models, while developing robust slide-level or patient-level aggregation method.

## 6 Conclusion

The proposed approach demonstrates potential as a clinical decision-support tool for HER2 immunohistochemistry assessment. By providing reproducible image-based scoring, it may help reduce inter-observer variability and support the identification of diagnostically challenging cases, particularly equivocal HER2 2+ cases that require confirmatory testing. Further prospective validation using multi-institutional datasets and correlation with ISH/FISH results is warranted to determine its clinical applicability in routine pathology workflows.

## Data Availability

The original contributions presented in the study are included in the article/supplementary material. Further inquiries can be directed to the corresponding authors.
